# Giant Congenital Hemangioma of the Skull: Prenatal Diagnosis and Multimodal Endovascular and Surgical Management

**DOI:** 10.3390/medicina60010145

**Published:** 2024-01-12

**Authors:** Andrea M. Alexandre, Andrea Romi, Simona Gaudino, Marco Gessi, Paolo Frassanito, Arianna Camilli, Scarcia Luca, Alessandro Pedicelli

**Affiliations:** 1 UOSA Interventional Neuroradiology, Fondazione Policlinico Universitario Agostino Gemelli IRCCS, 00168 Rome, Italy; andrea.alexandre@policlinicogemelli.it (A.M.A.); alessandro.pedicelli@policlinicogemelli.it (A.P.); 2 Neuroradiology Unit, IRCCS Policlinico San Matteo, 27100 Pavia, Italy; andrearomimd@gmail.com; 3 Neuroradiology Unit, Fondazione Policlinico Universitario Agostino Gemelli IRCCS, 00168 Rome, Italy; simona.gaudino@policlinicogemelli.it; 4 Neuropathology Unit, Fondazione Policlinico Universitario “A. Gemelli” IRCCS, Università Cattolica del Sacro Cuore, 00168 Rome, Italy; marco.gessi@policlinicogemelli.it; 5 Pediatric Neurosurgery, Fondazione Policlinico Universitario A. Gemelli IRCCS, Largo A. Gemelli, 8, 00168 Rome, Italy; paolo.frassanito@policlinicogemelli.it; 6 School of Medicine, Catholic University, 00168 Rome, Italy; aricamilli@gmail.com; 7 Department of Neuroradiology, Henri Mondor Hospital, 94000 Creteil, France

**Keywords:** congenital intraosseous hemangioma, endovascular treatment, fetal MR, neonatal neurosurgery, personalized medicine

## Abstract

*Introduction*: calvarial capillary hemangiomas are vascular tumors rarely seen in newborns. Differential diagnosis may be not straightforward on imaging studies and the management depends on patient and lesion characteristics. *Case report*: we present the case of a large congenital intracranial extra-axial lesion detected by routine prenatal US screening, a giant calvarial congenital hemangioma, treated with a multimodal strategy. Neonatal MR showed a hemorrhagic solid lesion, causing compression of brain tissue. Conservative treatment was attempted, but a one-month follow-up MR showed growth of the lesion with increased mass effect. Pre-operative endovascular embolization and surgical resection were performed. The pathology was consistent with intraosseous capillary hemangioma. The post-operative course was uneventful. At the 8-month follow-up, the patient had no clinical deficits and MR showed complete resection of the lesion. At the 13-month follow-up, the patient was asymptomatic, showing normal neurological examination and psychophysical development. *Conclusions*: although wait-and-see policy is feasible for small and asymptomatic lesions, radical resection is indicated when the mass is large, thus causing severe mass effect on the brain. Hypervascularization of the tumor may be responsible for hemorrhagic complications and severe anemia. On these grounds, endovascular treatment is feasible and effective to reduce hemorrhagic complications.

## 1. Introduction

Congenital hemangiomas (CHs) are benign vascular tumors fully developed at birth. The evolution of the disease is variable since major complications related to tumor size and/or location may occasionally occur. On the other hand, CHs may even regress rapidly [1].

Intraosseous hemangiomas are uncommon, accounting for only 0.7% of all bony neoplasms. However, the skull is the second most common site of intraosseous hemangiomas after the spine, and most calvarial hemangiomas are of the cavernous type [2,3]. They are usually solitary lesions that are most commonly found in the parietal bones, followed by the frontal bones [4]. Most lesions present between the second and fourth decades of life, with congenital occurrence being extremely rare [5]. Diagnosis based on imaging studies is not straightforward and may affect subsequent management, especially in newborns.

We herein present an extremely rare case of a giant calvarial CH diagnosed prenatally. Management issues and a multimodal strategy, involving endovascular embolization and surgical resection in the first month of life, are thoroughly discussed.

## 2. Case Report

### 2.1. Presentation and Clinical Characteristics

An asymptomatic 35-year-old woman underwent a routine obstetrical ultrasound (US) at 35 weeks of gestational age during her second pregnancy. The US identified a large right parietal tumor in a male fetus. No laboratory alterations were disclosed.

**Figure 1:**
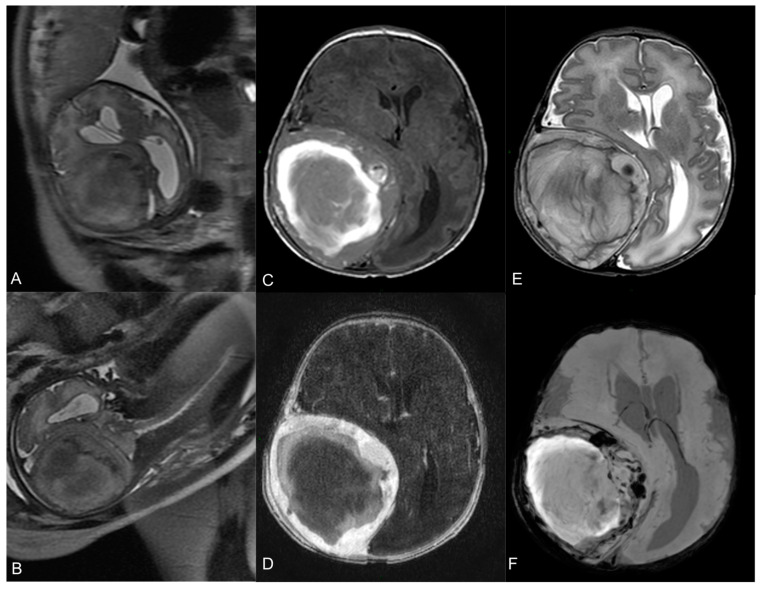
Fetal MR at 35-week gestational age—T2-w axial (**A**) and coronal (**B**) images show a large heterogeneous lesion (69 × 62 × 67 mm^3^) with severe mass effect on the surrounding brain. Neonatal pre-operative head MR performed 2 days after birth. Axial T1-w image (**C**) shows the hyperintense hemorrhagic component. Axial T2-w (**E**) demonstrates the mass effect on the right parietal lobe, with right lateral ventricle compression and contralateral midline shift (15 mm). Axial T1-w image after Gadolinium administration (**D**) demonstrates a peripheral solid part with intense enhancement. Axial SWI sequence (**F**) shows multiple hypointense spots within the lesion, indicative of hemosiderin.

A fetal magnetic resonance (MR) revealed an expansive extra-axial right fronto-parietal mass measuring 69 × 62 mm^2^, causing compression on the cerebral parenchyma and ventricular system, with a contralateral midline shift (14 mm), but without significative perilesional edema (Figure 1A,B). Visceral organs were normal.

Due to the compression of the brain parenchyma, delivery by cesarean section was scheduled one week later and performed without complications. At birth, the newborn presented a 37 cm head circumference with bulging but not tense anterior fontanelle, with neither neurological symptoms, hemodynamical instability, nor other major complications (birth weight was 2.6 kg, one-minute and five-minute Apgar scores were 8 and 10, respectively).

Subsequent MR confirmed the expansive, intracranial epidural lesion, with extension through the bone to the extracranial tissues, showing a slight dimensional growth since the fetal study. The lesion had a heterogenous signal on all sequences, a hemorrhagic central core and a peripheral, solid, contrast-enhancing component. Ipsilateral thickening of the dura was also noticed. Furthermore, a 4D time-resolved MR angiography showed arterial supply from a marked hypertrophic and high-flow right middle meningeal artery, with multiple flow-void images inside the mass, consistent with highly vascularized areas. There was no dilatated venous drainage. No anomalies of the cerebral parenchyma and myelinization were found (Figure 1C–E).

A wait-and-see policy was adopted.

### 2.2. Endovascular Embolization and Surgical Treatment

Medical treatment was considered, but the progressive dimensional growth of the mass required immediate treatment to avoid further complications.

At the one-month follow-up, in fact, a US demonstrated the growth of the lesion. Patient development was otherwise normal (body weight was 3.2 kg).

**Figure 2:**
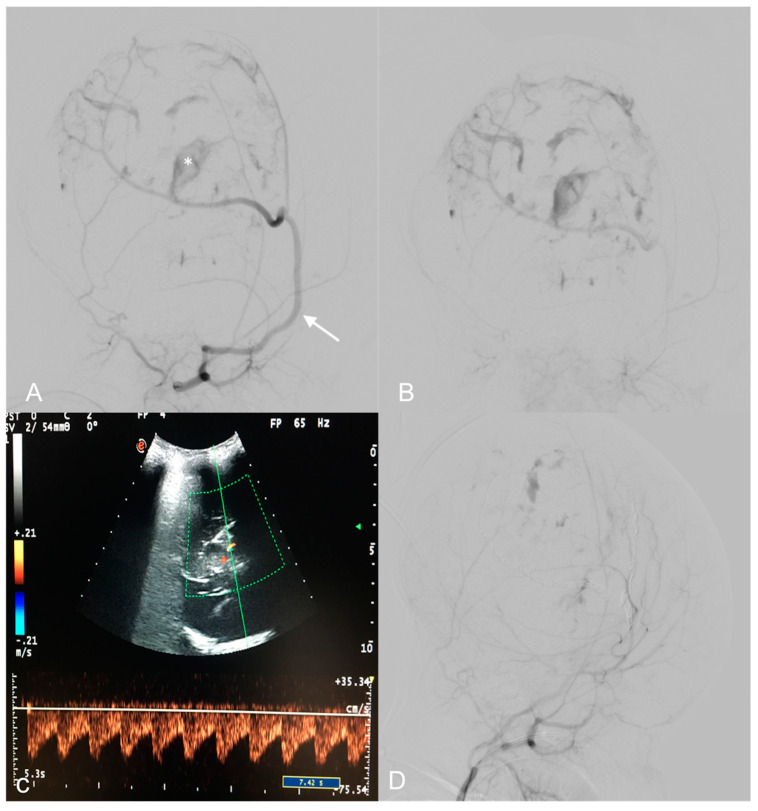
Pre-operative digital subtraction angiography (DSA) performed 1 month after birth (**A**,**B**)—Early and late acquisitions on right anterior oblique projections demonstrate arterial feeders, mainly from a hypertrophic right middle meningeal artery (arrow), minor feeders from the temporal and occipital ipsilateral arteries, and multiple intralesional blushes. A deep pseudoaneurysmatic formation (asterisk) was evident along the middle meningeal artery. US color Doppler at 7 days of life demonstrating a high, low-resistance arterial flow in an arterial feeder (**C**). Endovascular embolization with detachable coils and particles provided optimal devascularization of the lesion (**D**).

A Doppler US showed multiple high-flow and low-resistance feeding arteries (Figure 2C).

DSA revealed multiple arterial feeders arising from the superficial temporal artery and the occipital artery, and a main pseudoaneurysmal feeder from the middle meningeal artery. The lesion showed heterogeneous enhancement in its solid part. A trans-arterial embolization, using detachable coils and 150–255 nm polyvinyl particles, obtained a 90% lesion devascularization, useful in reducing surgical hemorrhagic risk (Figure 2A,B,D). The least possible amount of contrast medium was used according to the weight of the patient in order to avoid renal or hemodynamic complications. Radiation exposure was also maintained as low as possible during the embolization.

**Figure 3:**
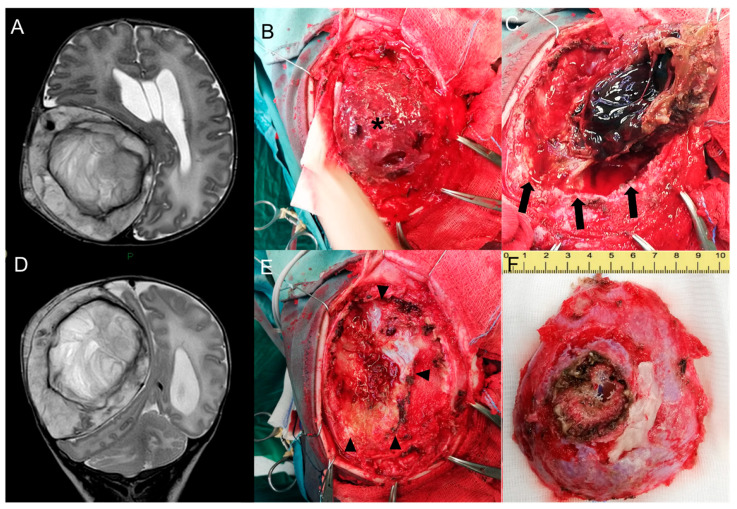
Pre-surgical head MR performed at 1 month—Axial (**A**) and coronal (**D**) T2-w images show growth of the lesion compared to the neonatal MR. Intra-operative pictures—Exposure of the lesion (asterisk) after subcutaneous dissection (**B**). Craniotomy enclosing the lesion and progressive dissection away from the dural plane (delimited by black arrows in Figure (**C**)) with preservation of the dural plane (black arrowheads, (**E**)) and macroscopical en bloc resection of the tumor (**F**).

Brain MR confirmed the lesion’s dimensional growth, with an increased contralateral shift of the midline and initial enlargement of the ventricular system. Posterior fossa structures, including the cerebellar tonsil, fourth ventricle, and vermis were caudally dislocated, with bilateral cerebellar edema (Figure 3A,D).

Surgical resection was completed the following day through a right fronto-parietal craniotomy enclosing the solid partially coagulated lesion, measuring 7.5 × 6 × 3 cm (Figure 3B,C,E,F).

The dural layer was intact at the end of the resection. Due to the young age of the patient and the preserved dural layer, the cranial repair was not completed. At the time of surgery, the patient weight was 3.3 Kg with an estimated blood volume of 260 mL. Blood loss was limited thanks to the pre-operative embolization, though requiring perioperative transfusion of red blood cells (70 mL). The patient was monitored in the pediatric intensive care unit for 72 h, requiring additional transfusion of red blood cells (70 mL), platelets (20 mL), plasma (45 mL), and AT-III (350UI). However, no hemodynamical complication was registered.

The following post-operative course was uneventful.

**Figure 4:**
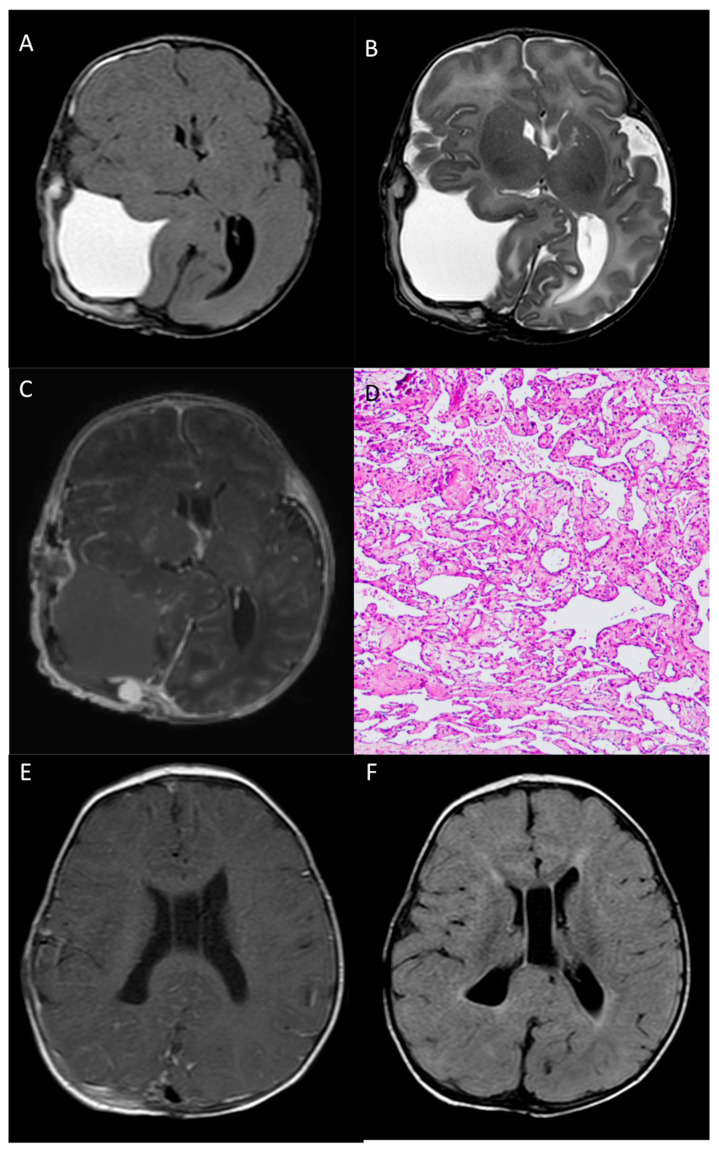
Post-operative head MR performed 2 days after surgery—Axial T1-w (**A**), axial T2-w (**B**) and post-contrast T1-w (**C**) images show gross total resection of the mass, with a minimal residual nodule next to the confluence of sinuses. Pathological examination (**D**) reveals, at the periphery of the hemorrhage, a vascular lesion composed of anastomosing, often dilated, capillary vessels (HE staining, 100× magnification). 8-month MR images (**E**,**F**) show complete resection of the lesion, with complete re-expansion of the parietal lobe.

The post-operative MR confirmed the radical resection of the mass, without complications. Cerebral and ventricular compression was markedly reduced, as well as the previously mentioned cerebral hernias. MR images clearly show the extra-axial location of the lesion (Figure 4A–C).

According to the histopathological analysis, the resected specimen was mostly composed of hemorrhage and fibrin material. At the periphery of the hemorrhage, a vascular lesion consisting of anastomosing and often dilated capillary vessels was present. This feature distinguishes capillary hemangiomas from cavernous hemangiomas, since the latter lack any mature vascular architecture and present dilated caverns. Scattered calcification and foci of papillary endothelial hyperplasia were also seen (Figure 4D).

At the 6-month follow-up, the patient was neurologically intact and in perfect clinical condition; 8-month MR images showed complete resection of the lesion, with complete re-expansion of the parietal lobe, no cortical anomalies of the cerebral parenchyma, and complete healing of the calvarial defect (Figure 4E,F).

At the 13-month follow-up, the patient was asymptomatic, showing normal neurological physical examination and psychophysical development. Thus, the bone defect was repaired with autologous left parietal bone.

## 3. Discussion

Vascular anomalies are categorized by the International Society for the Study of Vascular Anomalies into two types of lesions: vascular tumors and vascular malformations [1]. CHs are benign vascular tumors that have grown to their maximum size at birth and do not exhibit accelerated postnatal growth [2]. They can be subdivided into three subgroups: rapidly involuting congenital hemangioma (RICH), non-involuting congenital hemangioma (NICH), and partially involuting congenital hemangioma (PICH).

Hemangiomas are slow-growing benign neoplasms, represented by proliferating blood vessels. They are histologically classified as cavernous and capillary hemangiomas, usually presenting between the second and the fourth decades of life [5,6]. They can be related to syndromic conditions, such as Sturge–Weber or PHACES [7]. Hemangiomas may cause cosmetic issues along with serious complications such as high-output cardiac failure or progressive anemia, even in sporadic cases [8].

In newborns, the scalp is a common localization and soft tissue hemangiomas rarely cross tissue planes. Their typical presentation consists of a subcutaneous, tender red or blue swelling, variably painful to pressure, and tends to involute usually within the fifth year of life. On the other hand, intraosseous hemangiomas account for only 0.7–1% [9] of all primary bone neoplasms. In this context, the calvarial bone epicenter represents the second most common location, but intracranial involvement is exceedingly rare.

Diagnosis is hardly obtainable with imaging alone, thus requiring histological analysis. However, being high-vascularized lesions, their biopsy is not routinely performed, and the definitive diagnosis can be reached only through the histopathological examination on the surgical specimen. Differential diagnosis includes traumatic (e.g., cephalohematoma) and neoplastic lesion, such as osteoma and osteoblastoma, aneurismal bone cyst, sinus pericranii, ossifying fibroma, melanotic neuroectodermal tumor, giant cell tumor, sarcomas, intradiploic dermoids, and giant cell reparative granuloma [10]. In the present case, the unusual presentation, the dimensions, and the uncertain nature of the lesion and the mentioned clinical complications observed in the newborn imposed a radical first-line therapy.

In this context, congenital hemangiomas of the calvarium are extremely rare [2,11,12,13,14,15,16]. In most of the cases, CHs appear as a thickening of the skull bone, eventually associated with the double skull sign [11]. On these grounds, these pictures should be carefully differentiated from cephalohematoma, primarily. More rarely, calvarial CH presents as space-occupying lesion. In these anecdotal cases, prenatal diagnosis is feasible [12]. However, we could not draw a definitive conclusion concerning the intraosseous location of these two cases. In fact, in both cases, the radiological images may have been consistent with a scalp CH with the erosion of the outer skull table. Additionally, both cases showed spontaneous regression and, consequently, did not undergo surgical resection. Histological specimens of these cases were consistent with cavernous hemangioma, that is more frequently observed at this site (shown in Table 1).

**Table 1:** Summary of calvarial congenital hemangioma reported in the literature so far.

Authors, Year	n° Cases	Location	Radiological Aspect	Management	Histopathology
Yoshida et al., 1999 [16]	1	Lt Parietal	Skull bone thickening	Surgery	Cavernous
Koulouris et al., 2005 [9]	1	Multiple	Space-occupying lesions + Skull bone thickening	Prednisolone + Biopsy	n.a.
Elia et al., 2008 [12]	2	Rt occipital Lt frontal	Space-occupying lesion with extracranial extension Skull bone thickening	Spontaneous regression Spontaneous regression	n.a
Vural et al., 2009 [13]	1	Rt Parietal	Skull bone thickening	Surgery	Cavernous
Martìnez-Lage et al., 2010 [11]	1	Lt Parietal	Skull bone thickening	Surgery	Cavernous
Yucel et al., 2010 [14]	1	Rt Parietal	Skull bone thickening	Surgery	Cavernous
Rumana et al., 2013 [15]	1	Rt Parietal	Skull bone thickening	Surgery	Cavernous
Brichacek et al., 2018 [2]	1	Rt Parietal	Skull bone thickening	Biopsy + Propranolol	Cavernous
Benvenisti et al., 2014 [17]	1	Lt Occipital	Space-occupying lesion with intracranial extension	Biopsy + Propranolol	n.a.
Present study	1	Rt Fronto-Parietal	Space-occupying lesion with large intracranial extension	Endovascular Embolization + Surgery	Capillary

n.a. = not available.

On the contrary, the present case is the first capillary CH of the skull reported so far. Moreover, the radiological appearance was completely different from other congenital cases since the CH presented as a large extra-axial space-occupying lesion. In fact, the diploic space was largely swollen by the hemangioma with a predominantly intracranial extension, although the dural plane was not involved.

Surgical resection was performed in most of the cases. Other treatment options are propranolol [17], and systemic corticoids [18]. Thalidomide has been anecdotally reported as a treatment in cases of unresectable lesions [19], but never in infants.

In the present case, the giant size of the lesion and the mass effect secondary to the predominantly intracranial extension were in favor of surgical resection. On the other hand, the surgical and anesthesiologic risks due to the young age of the baby and the hypervascularized nature of the lesion drove the initial management to a conservative one followed by a strict follow-up to postpone surgery. Embolization at one month of age provided excellent devascularization of the tumor, thus reducing the intra-operative blood loss. Multidisciplinary discussion and multimodal management aiming to reduce blood loss is an essential point when dealing with such large congenital lesions in newborns and infants [20].

The peculiar feature of the present tumor, that did not tend to diffuse to different planes, allowed a safe dissection away from the overlying subcutaneous tissue and the underlying dura mater, that were not involved. Considering the preservation of the dura mater, the bone defect was left behind and repaired when the child had reached a greater physical development.

## 4. Conclusions

Congenital calvarial hemangiomas are rare vascular neoplastic lesions. Although in most of the cases, follow-up or conservative approaches are sufficient, they can occasionally cause symptoms or show locally aggressive behavior, thus requiring radical resection. Considering the large size and highly vascularized nature of the lesion, a combined therapeutic strategy with pre-operative endovascular embolization was safe and effective in reducing hemorrhagic complications and facilitating complete surgical resection.

## Data Availability

Data supporting reported results are available upon request.
